# Open source software security vulnerability detection based on dynamic behavior features

**DOI:** 10.1371/journal.pone.0221530

**Published:** 2019-08-23

**Authors:** Yuancheng Li, Longqiang Ma, Liang Shen, Junfeng Lv, Pan Zhang

**Affiliations:** 1 School of Control and Computer Engineering, North China Electric Power University, Beijing, China; 2 State Grid Information & Telecommunication Branch, Beijing, China; Victoria University, AUSTRALIA

## Abstract

Open source software has been widely used in various industries due to its openness and flexibility, but it also brings potential security problems. Therefore, security analysis is required before using open source software. The current mainstream open source software vulnerability analysis technology is based on source code, and there are problems such as false positives, false negatives and restatements. In order to solve the problems, based on the further study of behavior feature extraction and vulnerability detection technology, a method of using dynamic behavior features to detect open source software vulnerabilities is proposed. Firstly, the relationship between open source software vulnerability and API call sequence is studied. Then, the behavioral risk vulnerability database of open source software is proposed as a support for vulnerability detection. In addition, the CNN-IndRNN classification model is constructed by improving the Independently Recurrent Neural Net-work (IndRNN) algorithm and applies to open source software security vulnerability detection. The experimental results verify the effectiveness of the proposed open source software security vulnerability detection method based on dynamic behavior features.

## Introduction

In recent years, with the development of computer technology, especially the rise of Internet technology and related companies, open source software has greatly improved in terms of its performance, compatibility and user-friendliness. And its application in the operating system, compiler tool chain, database, Web server, mobile operating system and other aspects has become increasingly mature. Open source software has also brought amazing changes to the software industry with its openness and flexibility. However, with the open source software developing rapidly, it also brings us huge security problems [1–4]. From the beginning of 2015 to the beginning of 2017, the 360 security team selected 2228 open source projects from GitHub, Source forge and other open source communities for testing. The development languages involved include C, C++, C#, Java, etc. and the total number of detection codes was 257,835,574 lines. Then they found 2,626,352 source code defects and the overall average defect density of all test items was 10.19 every thousand rows. From the data obtained from the open source project testing program, the current security issues of open source software are very serious, and the security defect density in the code is high. According to NVD statistics, as of February 2017, there were more than 28,000 known security vulnerabilities related to open source software worldwide.

In view of the vulnerabilities and high risk characteristics of the open source software, it is necessary to conduct security analysis on open source software to determine whether it has security risks before referring to open source software. The current mainstream open source software vulnerability analysis technology is based on source code vulnerability analysis technology. Traditional vulnerability analysis techniques are divided into three types: static analysis, dynamic analysis, and dynamic and static combination analysis. There are some researches on static vulnerability detection [5–12]. In [5], the static analysis methods and other open source software source program analysis methods are analyzed, and the fact that static analysis methods have higher automation and faster speed is found and the static analysis methods have high false positive rate is also found. In [6], the static code attributes are extracted as machine learning features based on data flow analysis to identify vulnerabilities in open source software. In [7], DECAF implements a new instruction-level taint tracking engine at bit granularity, which exercises fine control over the QEMU Tiny Code Generator (TCG) intermediate representation to accomplish on-the-fly optimizations while ensuring that the taint propagation is sound and highly precise. In [8], the paper model the behavior of functions which have the stack overflow risks. Based on the model and taint analysis, the paper design and implement a method to detect stack overflow vulnerabilities. In [9], the paper focuses on software vulnerability static analysis techniques and tools. They first discuss the commonly-used static analysis techniques and tools, and compare these tools in a technical perspective, and then analyze the characteristics of these tools through the experiment. In [10], the paper developed a vulnerability detection tool that can help researchers and practitioners predict the future and unseen vulnerabilities in software, the tool collects metrics and defect data from various open source software projects and use the collected data as input of the defect prediction models. In [11], the paper present the results of evaluating multiple subsets of open source code for common software vulnerabilities using several static security analysis tools. There are also some researches on dynamic vulnerability detection on software [13–14] and dynamic and static combination analysis [15–17]. In [13], the paper design taint propagating algorithm based on data flow analysis and define several taint detection policies for security-critical function which used taint data in dangerous ways that could cause vulnerability exploit. Besides, a vulnerability exploit detection prototype system was implemented. In [14], a dynamic taint analysis method is proposed and the paper shows how control flow graph is used to analyze most type of programs. In [15], the approach combines symbolic execution with data flow analysis and static analysis, which allows a quick check of patch-related codes for automatically determining whether a patch brings new vulnerabilities. In [16], Targeting at PHP program, the paper proposes an SQL vulnerability detection method based on the injection analysis technology. The method implements the SQL vulnerability determination algorithm which is based on lexical feature comparison, on the basis of the combination of dynamic and static analysis technique. And the paper combines alias analysis technology, behavior model and SQL which is based on lexical feature comparison to design and establish a prototype system for SQL vulnerability detection. In [17], the paper presents a novel approach composing the static and dynamic analysis to integrate their advantages, and provides SDCF as a framework for thorough analysis of the target program. The framework translates the binary into assembly code and tracks the data flow. Then with static method, the system can get the important information which can’t be gained at runtime.

Although the current source code defect detection technology has been used in open source software security detection, most of the defect detection technologies for source code scan the open source software source code through lexical analysis, syntax analysis and other technologies. The main method is to verify whether the code meets the specifications, safety, reliability and other indicators, and the results are not completely credible. There are problems such as false positives, re-reports, and it is difficult to analyze complex defects [8]. Besides, these technologies are almost static analysis technology, and the dynamic behavior analysis technology is still very few application in defect detection. But the application of dynamic behavior analysis technology in malicious code detection is very extensive [18–21]. In [18], the idea that although malware and its variants may be very different from content signatures, they share some behavioral features at a higher level, which are more accurate in revealing the true intent of malware are proposed. The paper studies the malware behavior extraction technology, proposes a formal malware behavior features (MBF) extraction method, and proposes a malware detection algorithm based on malicious behavior features. In [19], the paper proposed a dynamic malware recognition method. The method uses a technique to explore multiple execution paths and trigger malicious behaviors and produce behavioral results. A unique set of malicious behavior and result correspondence (MBO) is constructed, and the weight of the malware family classification is determined. Then, the virtual monitor is used to explore multipath with the appropriate depth of detection to dynamically trigger malicious behavior. Finally, the triggered malicious behavior is modeled using the capabilities documented in the MBO to train the malware classifiers that identify unknown malware variants. In [20], a malware variant detection method based on behavior destructive features by combining static and dynamic analysis is proposed. In the method, the API calling sequence and the change of the process after the sample run is taken as the features. And they define the destructive features according to the API calling probability and process running frequency. And propose a fine-grained destructive features detection method for malware variant behavior. In [21], the paper design and implement a runtime behavior signature which can represent both the logic structures and the runtime behaviors of an app. The runtime behavior signature is effective to detect malware variants and transformed malware. They implement a lightweight, in-device malware detection system, for Android devices and propose two novel interception techniques, and show that it is easy to deploy and it provides informative alerts to users.

There is little research on applying behavior analysis technology to software vulnerability detection, combined with its application in malicious code detection, the paper proposes an open source software security analysis method based on dynamic features. Dynamic behavior analysis uses a debugger to trace software running process to analyze whether open source software really has security issues. By dynamically analyzing and extracting behavior information such as API sequences called during software running, and using a representative large number of software samples to construct a classification model, it is very useful and reliable in judging whether the software has security vulnerability.

Based on the further study of behavior features extraction and vulnerability detection technology, the paper proposes a method to use behavioral features to detect open source software vulnerabilities. The main contributions of the paper can be summarized as the following: First, we study the relationship between open source software vulnerabilities and API call sequences, and construct an open source software behavior vulnerability risk library, and use it as a support for vulnerability detection. Second, we construct the CNN-IndRNN classification model by improving the independent recurrent neural network (IndRNN) algorithm, and apply it to the open source software security vulnerability analysis. Finally, we carried out experiments, and the experimental results verified the method proposed in the paper has a good effect in detecting the security risk of open source software.

The rest of the paper is organized as follows: Section 2 introduces the dynamic behavior features extraction of open source software, the construction of the classification model CNN-IndRNN and vulnerability detection process based on dynamic behavior features. Section 3 shows our experimental results and the comparative analysis of the results. Finally, we conclude the paper in Section 4.

## Materials and methods

### Dynamic behavior features extraction of open source software

The extraction of dynamic behavior features of open source software is the key to analyze the security vulnerabilities of open source software. By analyzing the behavior of open source software during its operation, we can know the purpose of the software behavior and determine whether this behavior is risk or not. Through the behavior analysis of the open source software running process, and reference other papers, the paper divides the behavior of open source software in the running process into the following categories: registry behavior, file operation, network behavior, process operation, service behavior, HOOK behavior, information collection, function call behavior, guardian behavior, etc.

During the running process of the software in Windows, certain API functions must be called to implement these behaviors. Therefore, we can obtain the software behavior by monitoring the calls to such API functions during the running of the software. Some key API call sequences can expose the goal of software behavior, so we can track the call sequences of these API functions during dynamic analysis. These call information can be used to analyze the vulnerability of the software to determine whether its behavior is a security risk.

The paper obtains some software vulnerabilities and possible risk behaviors by studying CVE vulnerability database and other large vulnerability information databases, such as CWE, NVD, CNNVD, etc. Through comparative analysis, the software behavior security risk behaviors are roughly divided into 10 types: registry operation, network operation, process operation, file operation, thread management, memory management, equipment management, service management, interface management, and Hooking [22–23], and built a key API function call relationship corresponding to these risk behaviors, forming a library of open source software behavior risk vulnerabilities. Some API function calls in Windows are shown in Table 1:

**Table 1:** Software behavior and the corresponding key API function.

Software behavior	Key API functions
**Registry operation**	RegCloseKey, RegConnectRegistry, RegCopyTree, RegCreateKeyEx, RegDeleteKey, RegDeleteKeyEx, RegDeleteKeyValue, RegDeleteTree, RegDeleteValue, RegEnumKeyEx, RegEnumValue, RegGetValue, RegOpenKeyEx, RegQueryInfoKey, RegQueryValueEx, RegSaveKey, RegSetKeyValue, RegSetValueEx, NtOpenKey, NtQueryValueKey, NtCreateKey, NtDeleteKey, NtDeleteValueKey, NtNumberateKey, NtRenameKey
**Network operation**	DnsQuery, HttpOpenRequest, HttpSendRe-quest, InternetOpen, InternetReadFile, Inter-netWriteFile, InternetConnect, InternetOpenUrl, GetAddrInfo
**Process management**	CreateProcess, CreateProcessAsUser, ExitProcess, GetCurrentProcess, GetCurrentProcessId, GetCurrentProcessorNumber, OpenProcess, GetProcAddress, NtCreateSection, NtCreateUserProcess, NtOpenSection, NtWriteVirtualMemory, ReadProcessMemory, VirtualProtectex
**File operation**	CopyFile, CopyFileEx, CreateFile, DeleteFile, DecryptFile, EncryptFile, FindFirstFile, FindFirstStreamW, FindNextFile, GetFileSize, GetFileType, OpenFile, ReadFile, UnlockFile, CreateDirectory, CreateDirectoryEx, CreateDirectoryTransacted, RemoveDirectory, RemoveDirectoryTransacted
**Thread management**	CreateThread, ExitThread, GetThreadId, CreateRemoteThread, OpenThread, TerminateThread, ResumeThread, NtCreateThread, NtOpenThread, NtSuspendThread, NtSetContextThread, RtlCreateUserThread
**Memory management**	CopyMemory, FillMemory, MoveMemory, SecureZeroMemory, SetSystemFileCacheSize, VirtualAlloc2, VirtualAllocEx, VirtualFree, VirtualLock, VirtualUnlock, VirtualQuery
**Equipment management**	OpenService, OpenSCManager, StartService, DeleteService, CreateService, ControlService, GetServiceKeyName, DeviceIoControl
**Service management**	CreateService, DeleteService, OpenScmanager, OpenService, ControlService, StartService
**Interface management**	CloseSocket, Connect, Accept, Listen, WSARecvForm, WSASend, WSASendto, WSASocket, WSAStartup
**Hook**	CallMsgFilter, CallNextHookEx, SetWindowsHookEx, UnhookWindowsHookEx

Through the research on relationship of the open source software risk behavior and its corresponding key API function call, we get a list of key API functions that need to be monitored. By monitoring the API function call during the software running process, we extract the key API functions and call sequence, and vectorized these key API functions.

The paper uses Cuckoo Sandbox to monitor the running process of open source software, and obtains the calling information of various API functions in the running process. According to the behavior vulnerability database, the software behavior information is vectorized, and finally the behavioral feature matrix with dimension size 20*20 is obtained. Each type of behavioral feature consists of 40 elements, that is, two rows in the feature matrix, and 40 elements respectively correspond to 40 API functions in the behavior vulnerability database. When the software behavior calls this API function, the corresponding matrix element value will be set to the number of calls, and if it is not called, the value is set to 0. If the key API function of such type behavior is less than 40, the value is filled with -1.

### Construction of classification model CNN-IndRNN

The open source software vulnerability detection based on behavioral characteristics requires that the classification model can accurately determine whether the input software sample behavior contains risk behavior. Convolutional neural networks (CNN) have better classification ability in vulnerability detection, but can’t retain the calling relationship between API functions well, and the overly complex neural network structure will cause gradient disappear with the increase of the number of layers. Recurrent neural networks (RNN) are often used to deal with sequence problems, which can better express the call relationships between API functions. However, when the API call sequence is too long, RNN also has the problem of gradient disappear, and the neurons in RNN hidden layer are all entangled and their behavior is difficult to explain. Independent Recurrent Neural Network (IndRNN) is an improved model of RNN. It has a relatively simple internal structure. The neurons in each layer are independent of each other, but only cross-layer connections, and fewer parameters can make internal calculations less. And it is easier to converge.

**Fig 1:**
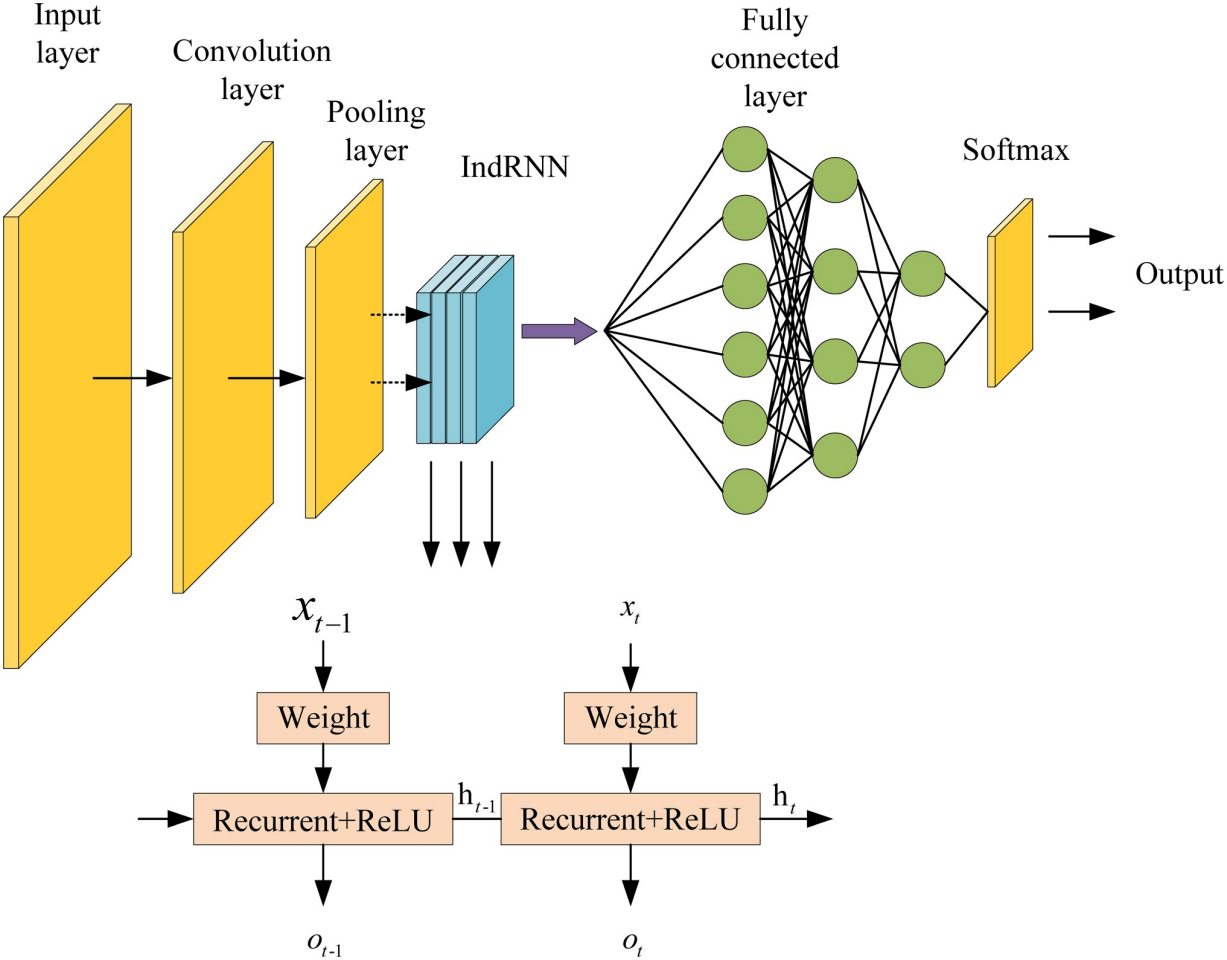
Structure diagram of CNN-IndRNN model.

By combining CNN and IndRNN models, the paper combines the advantages of the two models and constructs a new model named CNN-IndRNN, which is more suitable for vulnerability detection based on behavioral features. The CNN is used as an interface to interact with the feature vector, and the IndRNN is used as a module for processing the API function call sequence. The efficiency of CNN in processing data is higher and faster than that of RNN, and the automatic learning ability of convolution kernel is stronger than that of RNN. The IndRNN model not only solves the problem of gradient disappearance in CNN, but also captures the call sequence information between API functions neglect by CNN. The structure of the CNN-IndRNN model is shown in Fig 1.

The input layer of CNN can directly process multidimensional data to process high-dimensional data quickly and guarantee the invariance of feature data to the greatest extent when dimension reduction. And next structures are the CNN’s convolutional layer and pooling layer. Besides, the IndRNN is embedded between the pooling layer and the fully connected layer, and IndRNN is used to preserve the calling relationship between the API functions in the software behavior feature. Finally, the normalization process is performed through the fully connected layer, and the processed output value is sent to the softmax layer for classification detection to obtain a classification result.

Due to the use of gradient descent for learning, the input features of CNN need to be standardized. The input layer used in this paper is a pre-processed 20*20-dimensional feature matrix. The parameters of convolutional layer include convolution kernel size, step size and padding. The three parameters determine the size of the output feature map of the convolutional layer, which is an important hyper parameter of the convolutional neural network. The convolution kernel size can be specified as any value smaller than the input matrix size. The larger the convolution kernel is, the more complex of the extractable input features are. The convolution kernel size used in this paper is 4*4, and the scanning distance of the perceptual field of view to the input data is set to 2. There may be an out-of-bounds phenomenon when scanning to the boundary. The boundary needs to be expanded, and the value of the outbound part is set to zero. The input of the convolutional layer is a feature matrix of 20*20 in the input layer, and the matrix dimension of the output is determined by Eq (1):

$$\left\{ \begin{array}{l} height_{out}=\frac{height_{in}-height_{kernel}\text{+}2*padding}{stride}+1\\ width_{out}=\frac{width_{in}-width_{kernel}\text{+}2*padding}{stride}+1 \end{array}\right.$$ (1)

Where *height* and *width* represent the length and width of the feature matrix, *padding* is the fill mode, and *stride* is the step size. In the CNN-IndRNN model, *padding* takes 0 and *stride* takes 2, so the output matrix dimension is 9*9, that is, the matrix dimension of the convolutional layer output is 9*9. The pooling layer mainly performs compression and dimensionality reduction of features and prevents over-fitting. In the pooling layer, a filter with a size of 2*2 is used, and the step size is selected to be 1. The dimension of the matrix that can be derived from the pooling layer output is 8*8.

This paper embeds IndRNN between the pooled layer and the fully connected layer. Because CNN uses the convolutional layer and the pooling layer to process feature data, it often loses the up-and-down calling relationship between API functions in these behavioral features. In addition, the number of neural network layers is too easy to exist the gradient disappearance problem. So the IndRNN model is used to store the API function call information and solve the gradient disappearance problem in the whole model.

The structure of IndRNN is shown in Fig 1 [24], which can be expressed by Eq (2):

$$h_{t}=\sigma ({\text{W}}_{{\text{x}}_{t}}+u⊙h_{t-1}+b)$$ (2)

where the recurrent weight *u* is a vector, ⊙ represents the Hadamard Product (the element is defined as the product of the corresponding elements of the two matrices). In IndRNN, each neuron in the same hidden layer is not connected to other neurons, and neurons can be connected by superimposing two or more layers of IndRNN. For the *n*th neuron, the state of the hidden layer *h*_*h*,*t*_ can be obtained from Eq (3):

$$h_{n,t}=\sigma ({\text{w}}_{n}{\text{x}}_{t}+u_{n}h_{n,t-1}+b_{n})$$ (3)

where W_*n*_ and *u*_*n*_ are the *n*th row of the input weight matrix.

The gradient back propagation of each layer in the circulatory neural network over time, because the neurons in the same layer of network are not interconnected, the gradient in the IndRNN can be calculated separately for each neuron. For the *n*th neuron that ignores the deviation *h*_*n*,*t*_ = *σ*(W_*n*_x_*t*_ + *u*_*n*_*h*_*n*,*t*−1_), assuming that the target that attempted to minimize at the time interval *T* is *J*_*n*_, then the gradient returns to the time *t* can be expressed by Eq (4):

$$\frac{\partial J_{n}}{\partial_{h_{n,t}}}=\frac{\partial J_{n}}{\partial h_{n,T}}\frac{\partial h_{n,T}}{\partial h_{n,t}}=\frac{\partial J_{n}}{\partial h_{n,T}}\prod_{k=t}^{T-1}\frac{\partial h_{n,k+1}}{\partial h_{n,k}}=\frac{\partial J_{n}}{\partial h_{n,T}}\prod_{k=t}^{T-1}\sigma_{n,k+1}^{\prime }u_{n}=\frac{\partial J_{n}}{\partial h_{n,T}}u_{n}^{T-t}\prod_{k=t}^{T-1}\sigma_{n,k+1}^{\prime }$$ (4)

where $\sigma_{n,k+1}^{\prime }$ is the derivative of the element activation function.

In order to maintain the long-term memory function of the circulating neural network, the current state (at the time *t*) can still effectively influence the future state (at the time interval *T*) after a large time interval. Therefore, the gradient in the time interval *T* can be effectively propagated to the time *t*. By assuming that the minimum effective gradient is *ε*, the recursive weight range of the IndRNN neurons in order to maintain long-term memory can be obtained.

Specifically, to remember the length of the interval *T* − *t*, need:$\left|{\text{u}}_{n}\right|\in \left[\sqrt[{\left(T-t\right)}]{\frac{\varepsilon }{\prod_{k=t}^{T-1}\sigma^{\prime }{}_{n,k+1}}},+\infty \right]$, that is, In order to avoid the disappearance of the gradient of the neurons, the above constraints should be met.

In IndRNN, we choose to use ReLU as the activation function with a gradient of 0 or 1. Considering that short-term memory is also important for network performance, especially for multi-layered RNNs, the constraints on the weight range of the ReLU activation function can be relaxed to: $\left|{\text{u}}_{n}\right|\in \left[0,\sqrt[{\left(T-t\right)}]{\gamma }\right]$.

IndRNN is followed by a fully connected layer, which functions to connect all features, compress multiple Maps into one X-dimensional vector, and send the output values to the softmax classifier in the processing vector space.

### Vulnerability detection based on dynamic behavior features

The vulnerability detection process based on dynamic behavior features can be divided into two stages: code behavior features extraction and preprocessing, vulnerability detection model training and testing.

In the code behavior feature extraction and preprocessing stage, we first refer to the real vulnerability case in CVE, and extract the specific error API function in the case, and divide the API function into 10 categories, thus constructing the open source software vulnerability library. Then based on the API function in the vulnerability library, run the program, monitor the API function call during the running process, and extract the call sequences of the monitored API function.

In the training and testing stage of the vulnerability detection model, we construct a training model and a test model. The entire compilation process uses the Python language. Tag the test sample, the sample tag with the vulnerability is set to "0" and the sample tag without the vulnerability is set to "1". Taking 200 samples as training data, the experiment uses batch extraction to extract a fixed number of samples from the test sample each time, and trains the model through multiple iterations. In the test phase, the trained CNN-IndRNN model is used to test whether the test results of the model are the same as the actual results, and the vulnerability detection effect of the CNN-IndRNN model is tested.

**Fig 2:**
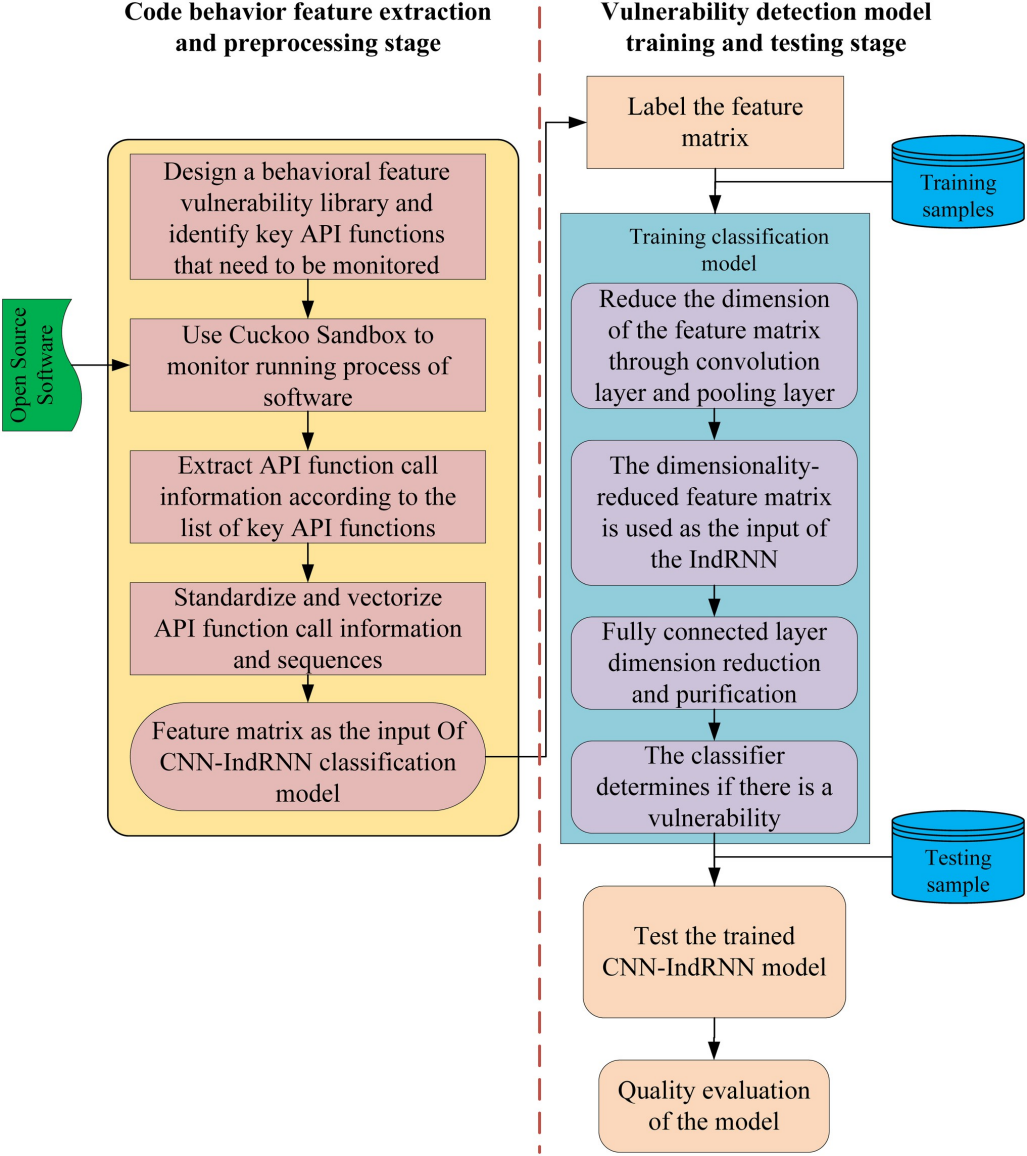
Open source software vulnerability detection process based on behavioral features.

The overall process of vulnerability detection of open source software based on behavior features is shown in Fig 2.

## Results and discussion

### Experiment setup and experiment data

The experimental platform for monitoring the software running process in the experiment is Ubuntu 64-bit, the memory size is 2G, and the experimental software is Cuckoo Sandbox 2.0.6. During the experiment, the software behavior analysis needs to connect a virtual machine to run the software. The virtual machine platform is Oracle VM VirtualBox, the operating system is Windows 7 64-bit, and the memory size is 1G.

The experimental platform for training and testing the vulnerability detection model is Windows10 64-bits, and the memory size is 8G.

The experimental data in this paper is from the official website of the National Institute of Standards and Technology (NIST) [25] and contains examples of vulnerabilities in various CWE standard libraries. In the paper, the software in the data set is monitored, the behavior information is preprocessed, the behavior features are extracted, and the behavior features are standardized and vectorized. Finally, 300 samples were formed as experimental data (160 positive samples and 140 negative samples), and there are 14 CWE IDs in the 140 negative samples, and the corresponding vulnerable systems are Windows, and there are 12 vulnerability types on these samples, such as, buffer overflow, memory leak. Besides, 200 samples were used as training data (110 positive samples, 90 negative samples), and the remaining 100 samples (50 positive samples, 50 negative samples) were used to verify the vulnerability detection effect of the CNN-IndRNN model.

### Model evaluation criterion

Before training and testing the model, it is necessary to give an evaluation index of the vulnerability detection model. Precision, Recall and Accuracy are frequently used indicators. The experimental results of the detection model are divided into the following four cases according to the difference between the prediction result, that is, the model determines whether the sample is vulnerable and the real result: TP: the prediction result is positive, the real result is positive; FP: the prediction result is positive, the real result is negative; FN: prediction result negative, the true result is positive; TN: the predicted result is negative, and the true result is negative. The calculation formula for each evaluation indicator is defined as Eq (5):

$$\left\{ \begin{array}{l} Precision=\frac{TP}{TP+FP}\\ Recall=\frac{TP}{TP+FN}\\ Accuracy=\frac{TP+TN}{TP+FP+FN+TN} \end{array}\right.$$ (5)

### Experimental results and analysis

**Fig 3:**
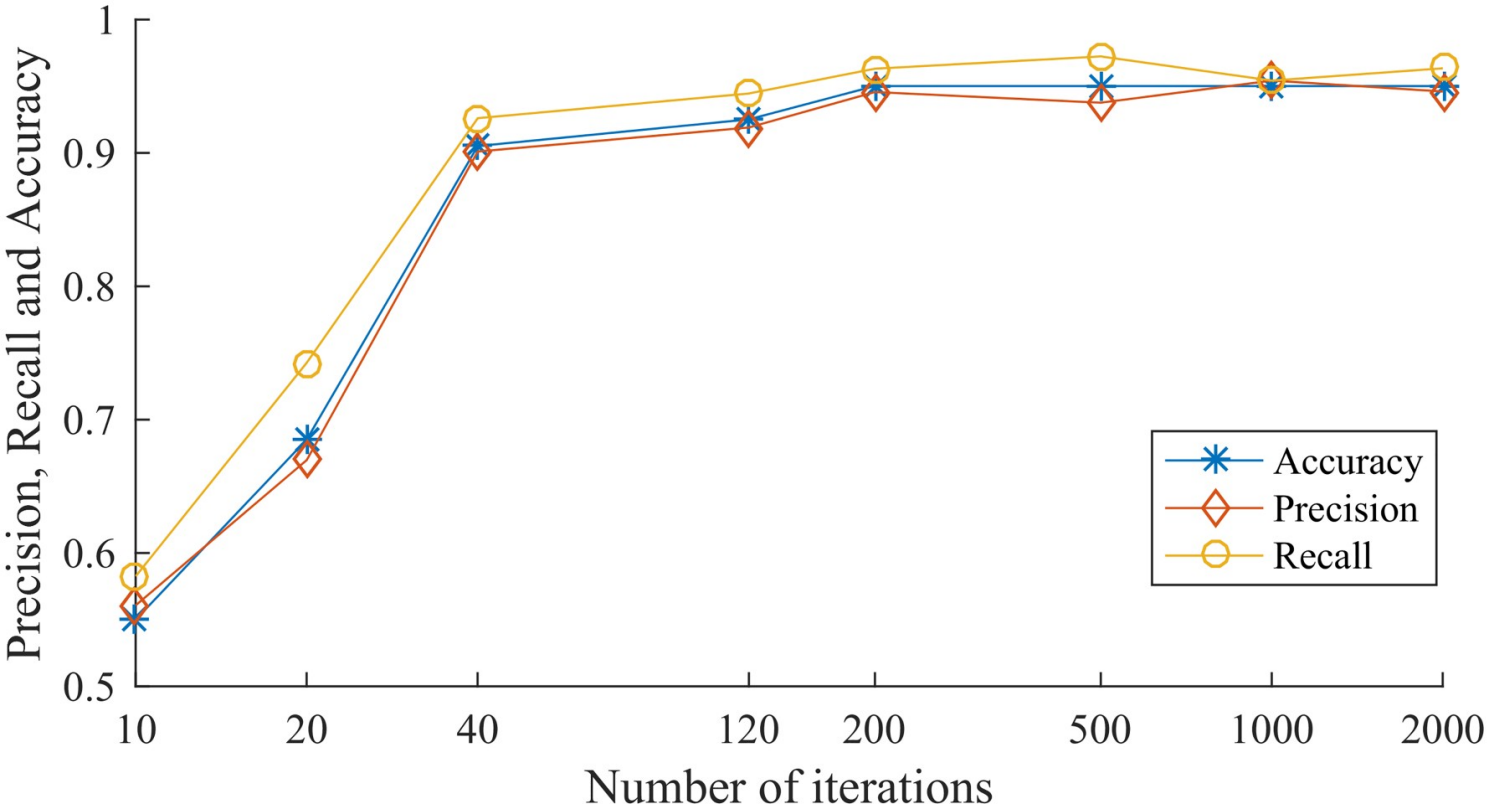
Training results of CNN-IndRNN model.

In the training phase, the batch gradient update algorithm (BGD) is used to update the gradients in batches. The training process is iterated 2000 times, and the current training Precision, Recall and Accuracy are output and the model document are saved after every 10 iterations. The model document saves the weight values of the settings when training the neural network, which is convenient for direct loading and using during the test phase. Part of the training results of the model are shown in Table 2 and Fig 3.

**Table 2:** Training results of CNN-IndRNN model.

Number of iterations	Precision	Recall	Accuracy
10	0.5607	0.5825	0.55
20	0.6696	0.7426	0.685
40	0.9009	0.9259	0.905
120	0.9189	0.9444	0.925
200	0.9455	0.963	0.95
500	0.9375	0.9722	0.95
1000	0.9541	0.9541	0.95
2000	0.9459	0.9633	0.95

It can be seen from the training results that as the number of iterations increases, the training Precision, Recall and Accuracy of the model increase. When the number of iterations reaches 200, the performance of the model reaches a maximum and remains almost constant. The Precision rate is about 95%, the Recall rate is about 96%, and the Accuracy rate is also 95%.

**Fig 4:**
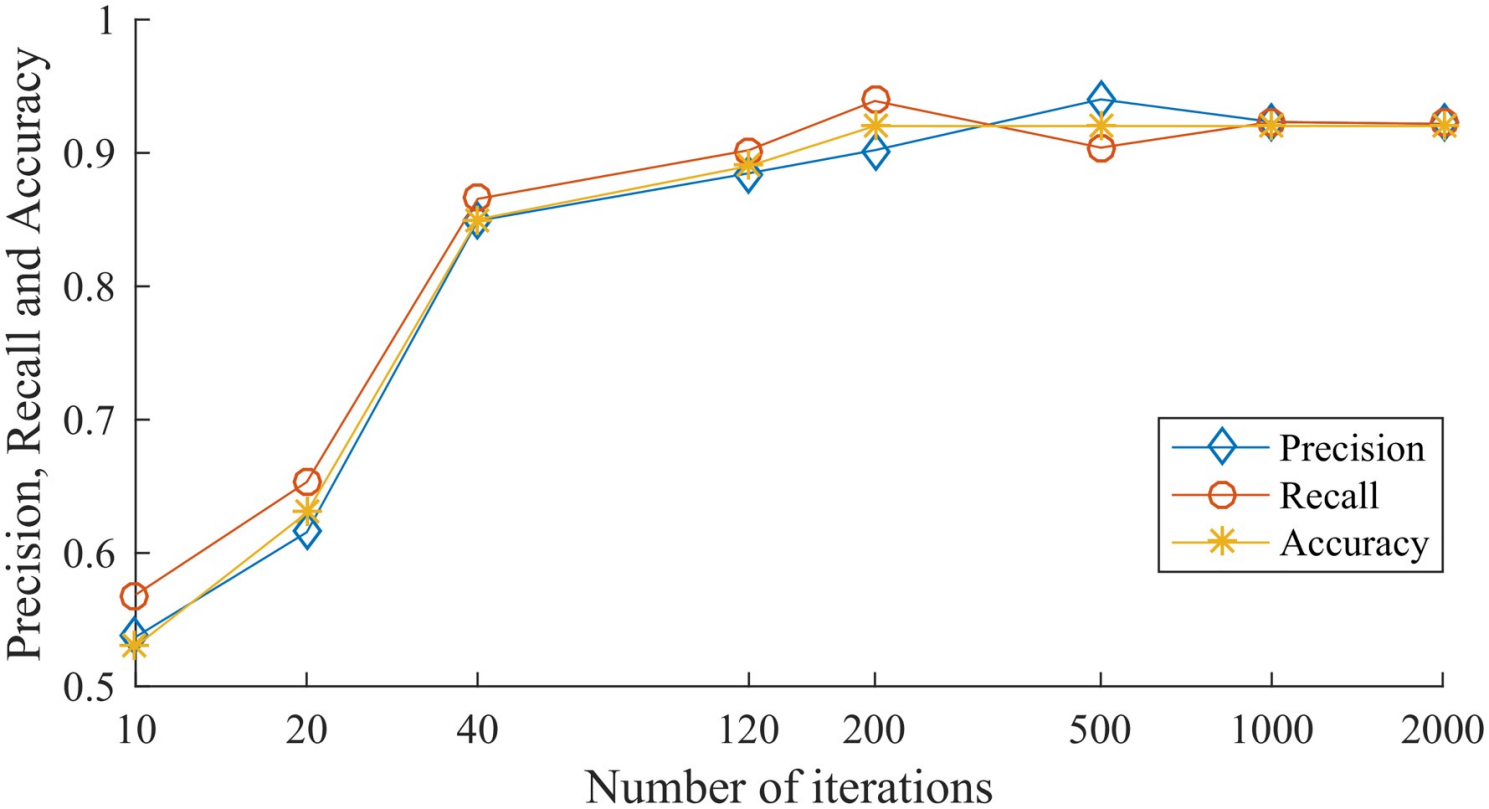
Test results of CNN-IndRNN model.

After the model is trained, the remaining 100 test samples are used to test the performance of the model. By directly utilizing the model document saved during training, the feature matrix of the test sample can be directly input into the trained model for vulnerability detection. The Test Precision, Test Recall, and Test Accuracy of the experimental results are shown in Table 3 and Fig 4.

**Table 3:** Test results of CNN-IndRNN model.

Number of iterations	Precision	Recall	Accuracy
10	0.573	0.5686	0.53
20	0.6154	0.6531	0.63
40	0.849	0.8654	0.85
120	0.8846	0.9019	0.89
200	0.9019	0.9388	0.92
500	0.94	0.9038	0.91
1000	0.9231	0.9231	0.92
2000	0.9216	0.9216	0.92

It can be seen from the experimental results that as the number of iterations increases, the performance of the model becomes better and better. After 200 iterations, the performance of the model is almost stable, and the Test Accuracy of the model reaches 92%.

**Fig 5:**
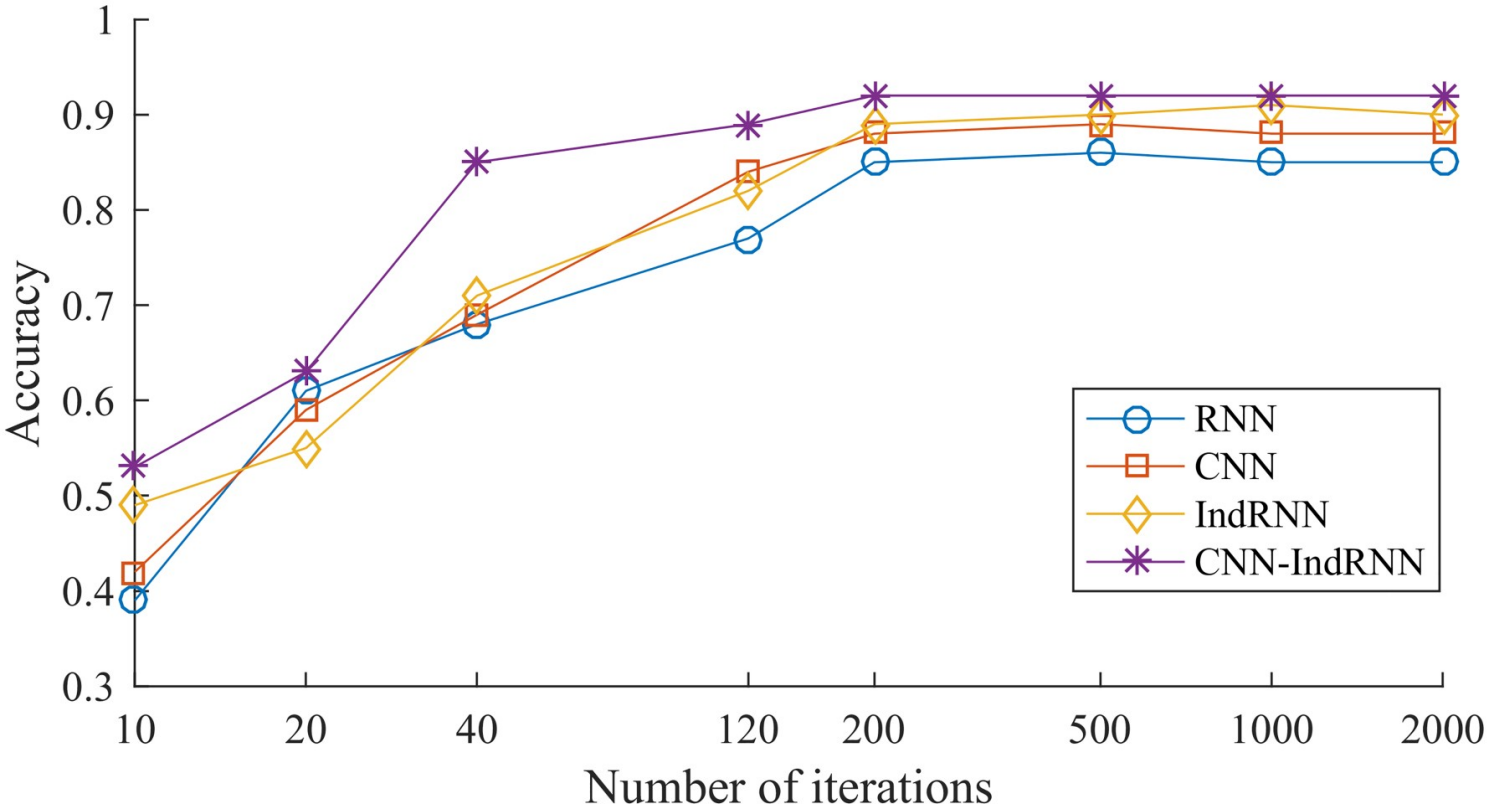
Test results comparison of models.

To further demonstrate that the CNN-IndRNN model has high vulnerability detection capabilities, the CNN-IndRNN model is compared with the CNN, RNN, and IndRNN. Using the same data set to train and test the CNN, RNN and IndRNN models, the experimental results of the four models were compared. During the training process, we counted the time cost of each model to iterate 2000 times, the results are shown in Table 4. And the Test Accuracy of each model is shown in Fig 5:

**Table 4:** Iteration time comparison of models.

Model	Time Cost of 2000 Iterations
CNN	653s
RNN	648s
IndRNN	637s
CNN-IndRNN	659s

From Table 4, we can see that the time iterated for 2000 iterations of the proposed model is almost equal to the other models, the most is about 3% slower, indicating that our proposed algorithm performs well in terms of speed. From Fig 5, we can know that the accuracy of CNN-IndRNN model is obviously the highest at 92%, followed by IndRNN (about 90%), CNN (about 88%) and RNN (about 85%).

The experimental results show that the proposed CNN-IndRNN model has better vulnerability detection performance. In terms of time consumption, it is almost equal to other models, but it is more accurate than the CNN, RNN, and IndRNN models. It is because of that the CNN-IndRNN model can retain the important features to the greatest extent in the feature matrix while reducing the dimension, and can preserve the behavior between the API functions.

## Conclusions

The paper proposes an open source software vulnerability detection method based on dynamic behavior features. The open source software is monitored, and dynamic behavior features information is extracted. According to the key API function list in the vulnerability library, the API function calls in the software running process are standardized and vectorized, and finally formed the features matrix. Besides, the paper combine the CNN model with the IndRNN model, proposes a new vulnerability detection model named CNN-IndRNN. The model can process high-dimensional feature data quickly and efficiently while retaining the call relationship between API functions, and the structure is simpler. The experimental results show that the CNN-IndRNN model has better vulnerability detection performance than the CNN, RNN and IndRNN models.
